# Inhibition of Doxorubicin-Induced Senescence by PPARδ Activation Agonists in Cardiac Muscle Cells: Cooperation between PPARδ and Bcl6

**DOI:** 10.1371/journal.pone.0046126

**Published:** 2012-09-25

**Authors:** Paola Altieri, Paolo Spallarossa, Chiara Barisione, Silvano Garibaldi, Anna Garuti, Patrizia Fabbi, Giorgio Ghigliotti, Claudio Brunelli

**Affiliations:** 1 Research Centre of Cardiovascular Biology, University of Genova, Genova, Italy; 2 Division of Cardiology, IRCCS University Hospital San Martino, University of Genova, Genova, Italy; 3 Laboratory of Cellular Therapies, Department of Internal Medicine, University of Genova, Genova, Italy; University of Sassari, Italy

## Abstract

Senescence and apoptosis are two distinct cellular programs that are activated in response to a variety of stresses. Low or high doses of the same stressor, i.e., the anticancer drug doxorubicin, may either induce apoptosis or senescence, respectively, in cardiac muscle cells. We have demonstrated that PPARδ, a ligand-activated transcriptional factor that controls lipid metabolism, insulin sensitivity and inflammation, is also involved in the doxorubicin-induced senescence program. This occurs through its interference with the transcriptional repressor protein B cell lymphoma-6 (Bcl6). Low doses of doxorubicin increase the expression of PPARδ that sequesters Bcl6, thus preventing it from exerting its anti-senescent effects. We also found that L-165041, a specific PPARδ activator, is highly effective in protecting cardiomyocytes from doxorubicin-induced senescence through a Bcl6 related mechanism. In fact, L-165041 increases Bcl6 expression via p38, JNK and Akt activation, and at the same time it induces the release of Bcl6 from PPARδ, thereby enabling Bcl6 to bind to its target genes. L-165041 also prevented apoptosis induced by higher doses of doxorubicin. However, while experiments performed with siRNA analysis techniques very clearly showed the weight of Bcl6 in the cellular senescence program, no role was found for Bcl6 in the anti-apoptotic effects of L-165041, thus confirming that senescence and apoptosis are two very distinct stress response cellular programs. This study increases our understanding of the molecular mechanism of anthracycline cardiotoxicity and suggests a potential role for PPARδ agonists as cardioprotective agents.

## Introduction

Anthracyclines are among the most effective anticancer treatments ever developed, but their clinical use is limited by their cumulative dose-related cardiotoxicity which may ultimately lead to a severe form of cardiomyopathy [1]. Despite solid evidence proving the induction of apoptosis in cardiomyocytes exposed to doxorubicin *in vitro*, there is controversy over whether apoptosis contributes to doxorubicin-induced cardiotoxicity *in vivo* [2]. It has recently been suggested that senescence may be a novel mechanism of cardiotoxicity induced by low doses of doxorubicin [3], [4]. Senescence is a fundamental cellular program that contributes to the physiology of living tissues, the aging process, and diseases [5]. Stress-induced premature senescence is the result of changes in the expression levels of many proteins that regulate cell cycle, cytoskeletal function and cellular architecture, and it leads to the impairment of cell functions, including the regenerative capacity [6]–[8]. The signal transduction pathways of the anthracycline-induced senescence program are not fully understood. There is however convincing evidence that p38 activation and expression levels of Telomere Binding Factor 2 (TRF2) play an important role [4], [9] Peroxisome proliferator-activated receptor δ (PPARδ) belongs to the nuclear hormone receptor superfamily together with PPARα and PPARγ [10]. PPARδ are ligand-activated transcriptional factors that regulate the expression of specific target genes involved in lipid metabolism, insulin sensitivity, energy homeostasis, obesity, and inflammation [11]–[15]. Activation/repression of target genes occurs via two molecular mechanisms: transactivation and transrepression. In the transactivation mode these nuclear receptors control gene expression by binding to a PPAR responsive element after heterodimerization with a retinoid X receptor. The transrepression activity of PPARs occurs through the physical interaction with other transcription factors. It has been shown that unliganded PPARδ sequesters the transcriptional repressor protein B cell lymphoma-6 (Bcl6) and prevents it from binding to the response elements in the promoter regions of its target genes. Following ligand binding, Bcl6 is released from PPARδ and inhibits inflammatory signals [15]–[17]. Bcl6 inhibits chemokine gene transcription in most tissues and cell types [18], regulates cell cycle progression [19], and is involved in lymphocyte activation and differentiation [20]. In the light of its effects on metabolism and inflammation, PPARδ activation has been seen as a promising approach for the treatment of atherosclerosis [21]. A number of studies using transgenic approaches and pharmacological interventions have shown that PPARδ also plays a crucial role in cardiomyocyte growth and survival, thus suggesting that PPARδ activation may be a therapeutic target in heart diseases [22]. Moreover, two studies demonstrated that PPARδ and Bcl6 may play a role in the regulation of cellular senescence [23], [24].

In this study we demonstrate that L-165041, a PPARδ agonist, is a cardioprotective agent that prevents senescence and apoptosis induced by low and high doses of doxorubicin, respectively. We prove that Bcl6 and Bcl6:PPARδ interference plays a central role in the regulation of senescence in cardiac muscle cells, and that the protective effects of the PPARδ agonist involve Mitogen-activated protein kinases (MAPKs) and Akt activation.

## Results

### Pre-treatment with the PPARδ Agonist Prevents the Prosenescent Effects of Doxorubicin 0.1 µM in Neonatal Rat Ventricular Myocytes and H9c2

**Figure 1:**
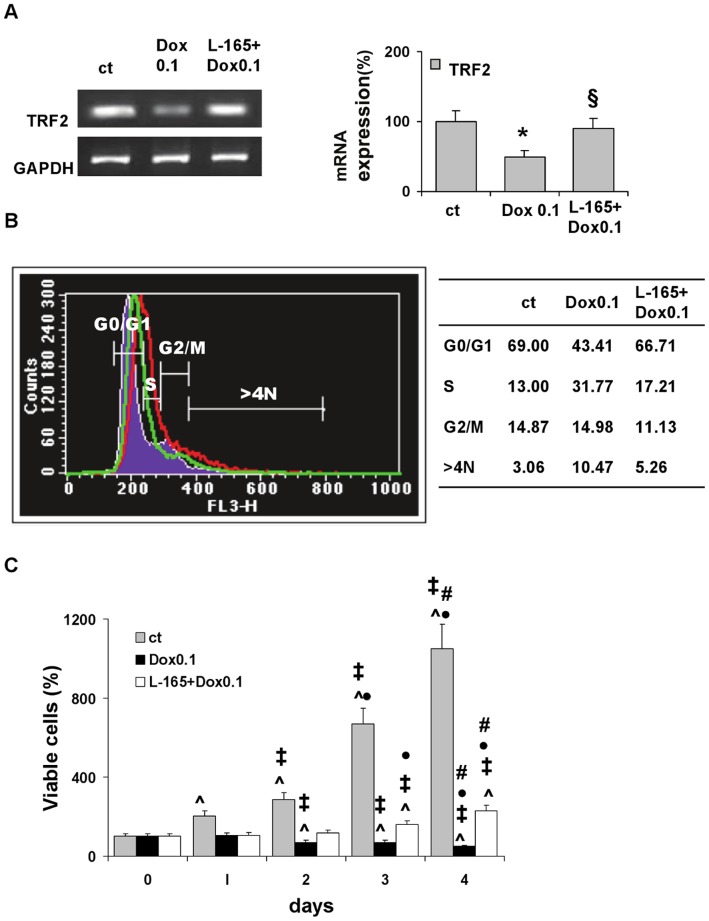
L-165041 prevents doxorubicin-induced changes in neonatal rat cardiomyocytes. Incubation for 3 h with low dose doxorubicin 0.1 µM (Dox 0.1) modifies the expression levels of telomere binding factor 2 (TRF2), the cell cycle and cell viability. Pre-treatment for 2 h with L-165041 (L-165) prevents doxorubicin-induced modifications. (A) TRF2 mRNA evaluated 4 h after doxorubicin treatment. *p<0.05 versus control (ct), §*p*<0.05 versus Dox 0.1. (B) FACS analysis of cell cycle distribution 24 h after treatment with Dox 0.1. Filled line: untreated cells; red line: cells treated with Dox 0.1; green line: cells pre-treated with L-165041 and then treated with Dox 0.1. Percentages of cell subpopulations are summarized in the Table. (C) Cell viability determined by MTT Assay. ˆ*p*<0.05 versus day 0. ‡*p*<0.05 versus day 1. • *p*<0.05 versus day 2. # *p*<0.05 versus day 3.

**Figure 2:**
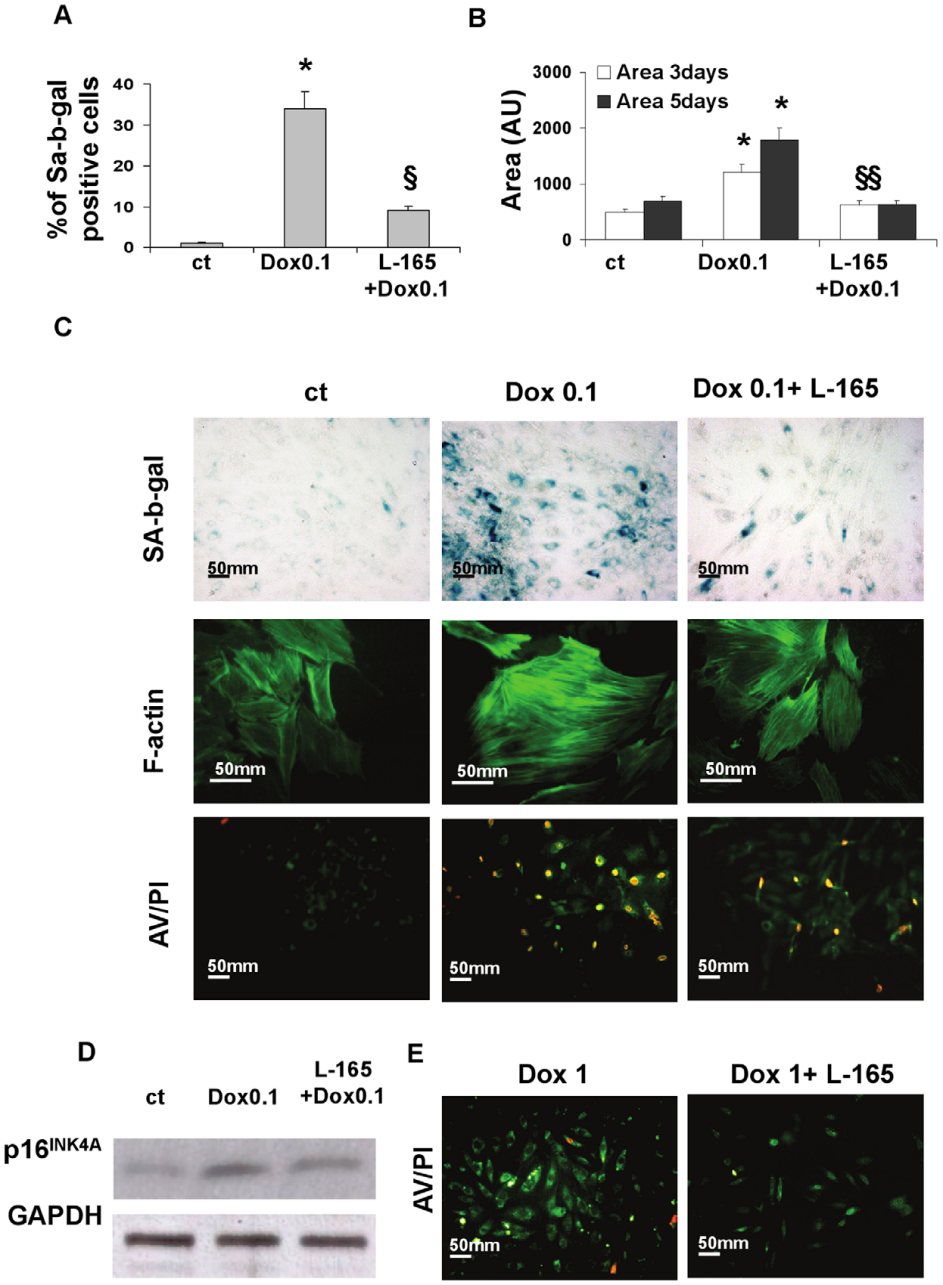
L-165041 prevents both senescence and apoptosis induced by doxorubicin. Pre-treatment with the PPARδ agonist L-165041 (L-165) prevents both senescence induced by low (0.1 µM) doses of doxorubicin (Dox) (A,B,C), and apoptosis induced by high (1 µM) doses of Dox (E) in neonatal rat cardiomyocytes. (A) Percentage of SA-b-gal positive cells 3 days after treatment. *p<0.05 versus control (ct), §*p*<0.05 versus Dox 0.1. (B) Cell size, 3 and 5 days after treatment. *p<0.05 versus ct, §*p*<0.05 versus Dox 0.1. (C) Photographs showing cells (from top to bottom): SA-b-gal activity evaluated 3 days after treatment with Dox 0.1 (magnification, ×200), F-actin density (magnification, ×400), and AV/PI staining evaluated 24 h after treatment with Dox 0.1 (magnification, ×200). (D)Western blot analysis of p16 INK4a evaluated 24 h after treatment with Dox 0.1 µM. (E) AV/PI staining evaluated 24 h after treatment with Dox 1 (magnification, ×200).

Previous studies have shown that brief exposure to low (0.1 µM) or high (1 µM) doses of doxorubicin induces either senescence or apoptosis, respectively, in neonatal rat ventricular myocytes. We examined the effects of pre-treatment with the PPARδ agonist L-165041 on neonatal cardiomyocytes exposed to a low, prosenescent dose of doxorubicin (Figs. 1 and 2).

Since previous studies demonstrated that down regulation of TRF2 (a nuclear protein that governs chromosomal stability) is at the core of the pathways that lead to doxorubicin-induced premature senescence [4], [25], we first examined the expression levels of TRF2. TRF2 maintains the telomere t-loop “endcapping” structure, thus preventing chromosome end-to-end fusion and chromosomal abnormalities. We found that doxorubicin down-regulates TRF2 (Fig. 1A), causes cell cycle alterations by increasing both the S phase and the hyperploid (>4N DNA) cell population (Fig. 1B), and also blocks cell proliferation (Fig. 1C). Pre-treatment with L-165041 prevented TRF2 downregulation, partially restored the cell cycle, and partially rescued the blocking of cell proliferation, (Figs. 1A, 1B, 1C).

Doxorubicin 0.1 µM also induced a senescence-like phenotype characterized by enzymatic SA-β-gal activity expression at pH 6.0 (Figs. 2A and 2C) as well as by an increase in size and a change in shape of the cells which became flatter (Figs. 2B and 2C). These changes were accompanied by increases in both the length and density of the cytoplasmic actin fibers, as evaluated by phalloidin staining, and by the early loss of cytoplasmic membrane integrity, as documented by Annexin/Propidium double staining (Fig. 2C). In fact, 24 hours after a brief incubation with doxorubicin, the majority of annexin positive cells were also propidium positive. This double positivity is predictive of late death for mitotic catastrophe in cells treated with low doses of doxorubicin and is in contrast with the typical pattern of early-stage apoptosis that is present in cells treated with pro-apoptotic doses and which is characterized by annexin positivity and propidium negativity (Fig. 2E).

Pre-treatment with the PPARδ agonist L-165041 lowered the increase in SA-β-gal activity and significantly attenuated all the cell morphology and structural changes induced by the exposure to low (Figs. 2A, 2B, 2C) and high doses of doxorubicin (Fig. 2E). We also evaluated the effects of doxorubicin 0.1 µM on p16INK4A, a cyclin-dependent kinase inhibitor thought to be a senescence–associated marker. Western blot analysis documented that doxorubicin induces changes in p16INK4A expression levelsand that L-165041 inhibits the increase of doxorubicin-induced p16INK4A (Fig. 2D).

**Figure 3:**
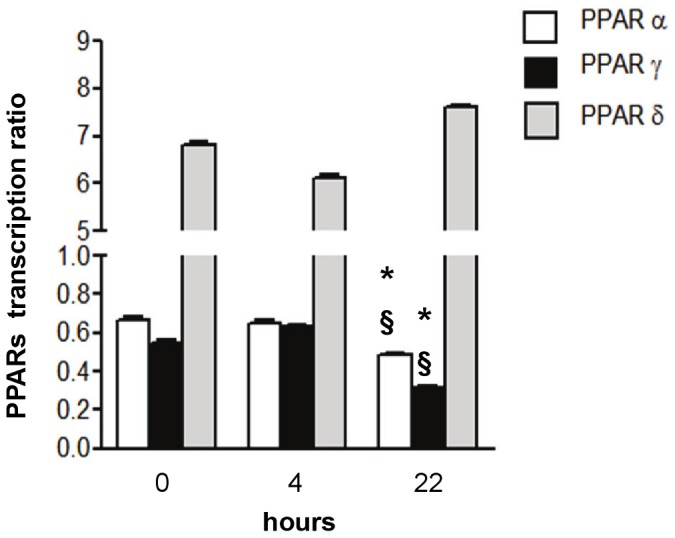
Effects of L-165041 on the expression levels of PPAR isoforms. Effects of L-165041 on PPARα, PPARγ and PPARδ evaluated in neonatal cardiomyocytes 4 h and 22 h after L-165041 treatment. *p<0.05 versus time 0, §*p*<0.05 versus 4 h.

Even though L-165041 is thought to be a specific ligand for the delta isoform which is the most highly expressed in the heart, we were interested in evaluating whether the obtained results could be in part attributed to the other isoforms. To this aim, we performed a quantitative Real Time PCR analysis (RTPCR) which demonstrated that PPARδ are much more highly expressed in neonatal cardiomyocytes than PPARα and PPARγ. The cells were treated for two hours with L-165041 and analyzed at 4 and 22 hours after the treatment. At 22 hours, L-165041 decreased the transcription ratios of PPARα and PPARγ and did not significantly increase the transcription ratio of PPARδ (Fig. 3).

After having carried out studies on neonatal cardiomyocytes, we performed experiments on H9c2 cells and obtained similar results (data not shown). H9c2 cells abundantly express the PPARδ subtype, where PPARα is mildly expressed and PPARγ is undetectable. Therefore, these cells represent a suitable model to investigate the role of PPARδ activation without the potential interference of other PPAR subtypes [26]. In the following paragraphs we report data collected from the experiments on H9c2.

### MAPK-mediated Signal Transduction Pathways Play a Key Role in the Cytoprotective Effects of the PPARδ Agonist L-165041 in H9c2 Cells

**Figure 4:**
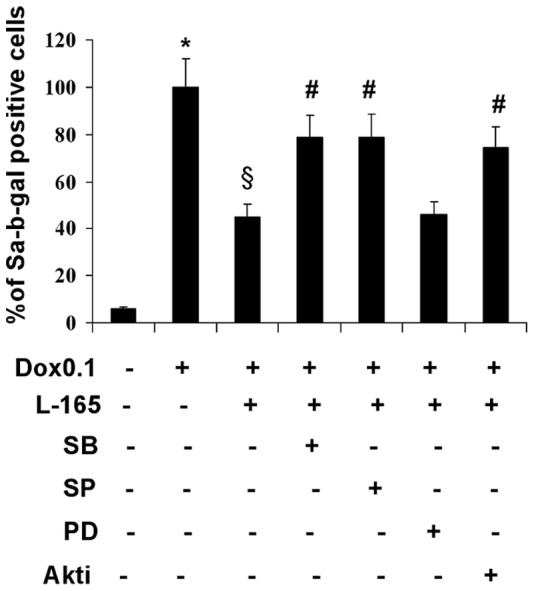
Pre-treatment with p38, JNK and Akt inhibitors prevents the anti-senescent effects of L-165041. The specific inhibitors of p38 (SB203580, SB), JNK (SP600125, SP), Akt (Akt1/2 kinase inhibitor, Akti) but not the inhibitor of ERK1/2 (PD98059, PD) reverse the effects of L-165041 (L-165) on doxorubicin-induced SA-b-gal activity. *p<0.05 versus untreated cells, §*p*<0.05 versus doxorubicin (Dox 0.1), # *p*<0.05 versus L-165+Dox 0.1.

In order to analyze which signaling pathways influence the protective effects exerted by L-165041, we blocked p38, JNK, Akt, ERK1/2 signaling by using the specific inhibitors SB203580, SP600125, Akt1/2 kinase inhibitor, and PD98059, respectively. Cells were assayed for SA-b-gal activity. Pre-incubation with the ERK inhibitor did not influence the protective effects of L-165041. In contrast, the effects of L-165041 on doxorubicin-induced SA-b-gal activity were attenuated by p38, JNK and Akt inhibition (Fig. 4). These results show the importance of p38, JNK and Akt signaling pathways in the cytoprotective effects of the PPARδ agonist L-165041 against the pro-senescent effects of doxorubicin 0.1 µM in H9c2 cells.

**Figure 5:**
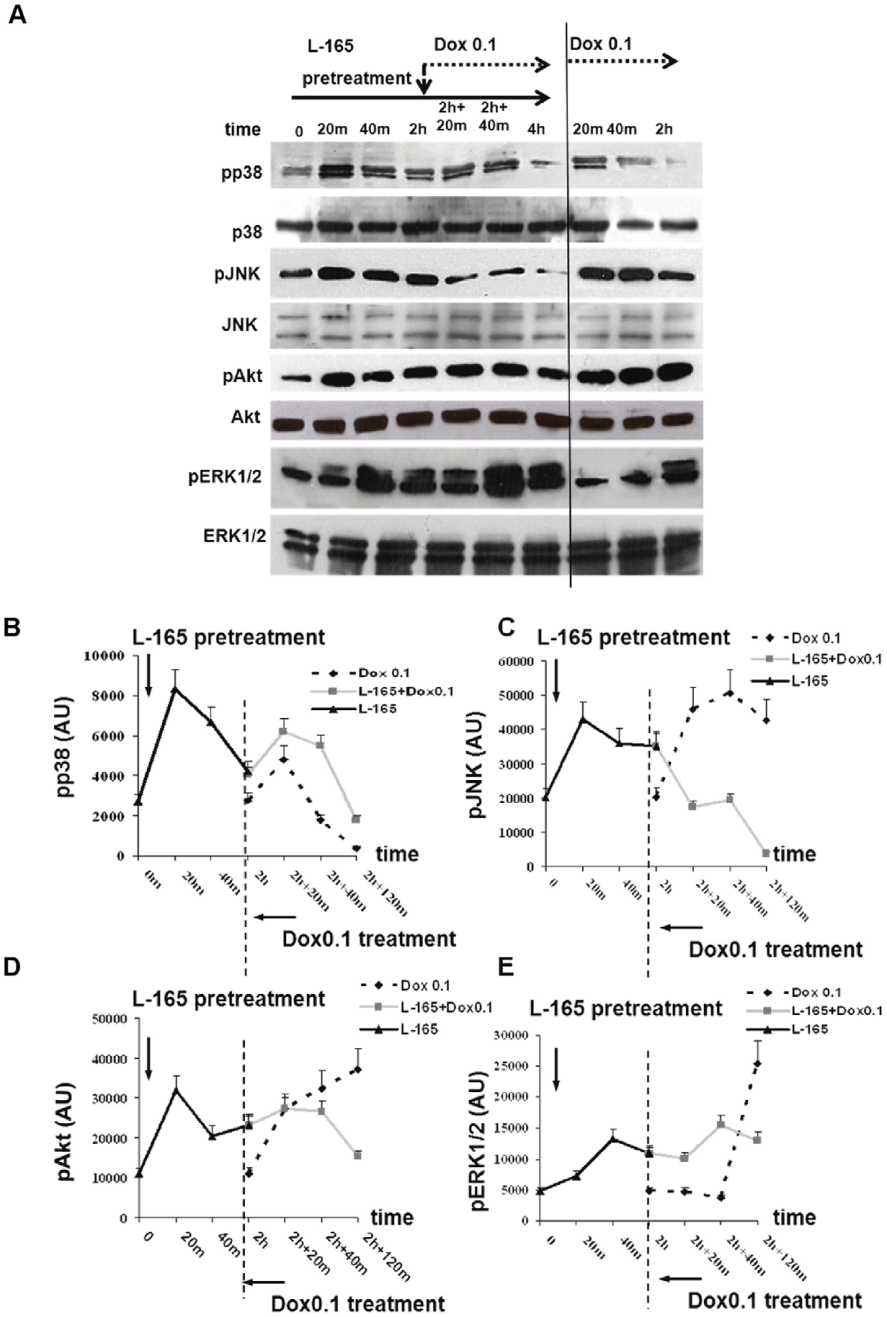
Effects of L-165041 and/or doxorubicin 0.1 µM on MAPKs and Akt phosphorylation. Both L-165041(L-165) and doxorubicin (Dox) 0.1 µM activate MAPKs and Akt. If cells are pre-treated with L-165, the doxorubicin-induced increase of pJNK and pAkt levels is inhibited, pERK levels are maintained sustained, while pp38 levels result higher than those induced by Dox 0.1 alone. (A) Time curve analysis of phosphorylated pp38, pJNK, pAKT, pERK1/2 and total p38, JNK, AKT, ERK1/2 evaluated by western blot. (B, C, D, E) Graphs showing values for pp38, pJNK, pAKT and pERK1/2 normalized to the amount of total enzyme.

These findings prompted us to investigate the effects of pre-treatment with L-165041 on doxorubicin-induced MAPK activation. To this aim, we first examined the effects of doxorubicin 0.1 µM given alone for 120 minutes. Figure 5 shows that doxorubicin induced an early increase in pp38, pJNK and pAkt levels, while an increase in pERK levels was observed 120 min after exposure to doxorubicin. We then examined the effects of L-165041 given alone. We found that L-165041 increased pp38, pJNK, pERK, and pAkt levels. Finally, we examined the effects of the sequential treatment with L-165041for 2 hours followed by the treatment with doxorubicin for 2 hours. It is interesting to note that the doxorubicin-induced changes in MAPK and Akt activation were influenced by pre-treatment with the PPARδ agonist L-165041. In fact, pre-treatment with L-165041 prevented the doxorubicin-induced increases in pJNK, pAkt and pERK levels and led to higher doxorubicin-induced pp38 levels as compared to the levels that were reached by doxorubicin alone. No changes in total MAPK and Akt protein levels were found (data not shown).

### Doxorubicin Increases PPARδ Protein Expression and Sequesters the Transcriptional Repressor Bcl6 in Unliganded PPARδ, while L-165041 Treatment Increases Both PPARδ and Free Bcl6 in H9c2 Cells

**Figure 6:**
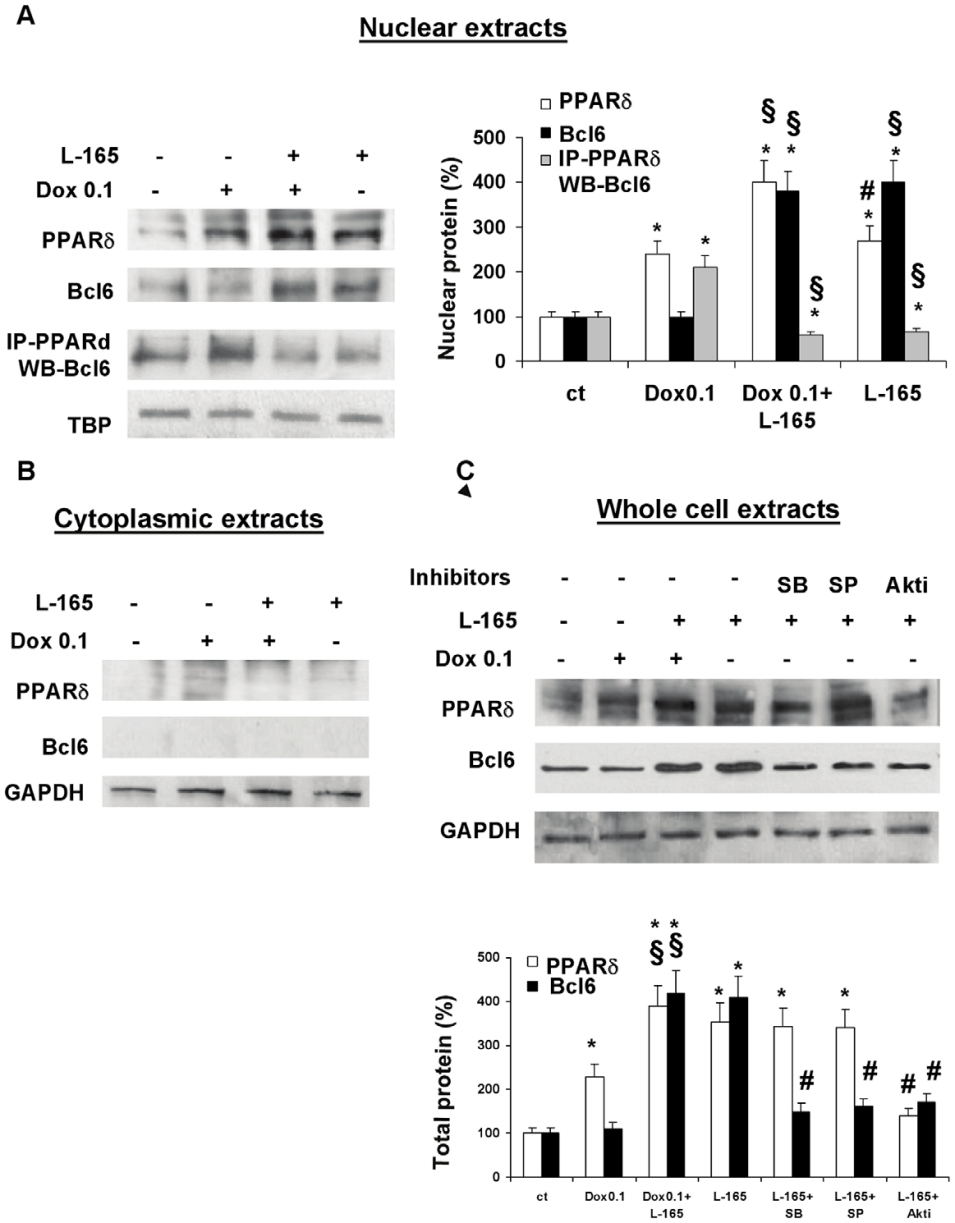
L-165041 and doxorubicin regulate PPARδ and Bcl6 expression and interaction: the role of MAPKs and Akt. H9c2 cells were pre-treated with or without L-165041 (L-165) for 2 h, then treated with or without doxorubicin (Dox) 0.1 µM for 3 h and analyzed after 24 h. Dox 0.1 increases PPARδ levels and enhances the interaction between Bcl6 and PPARδ but does not modify the amount of Bcl6. L-165041 increases the protein levels of both PPARδ and Bcl6 and induces the release of Bcl6 from PPARδ. Cytoplasmic extracts reveal nuclear localization of PPARδ and Bcl6. (A) Nuclear extracts were assayed by western blot analysis with PPARδ or Bcl6 antibodies; co-immunoprecipitation of PPARδ and Bcl6 (IP-PPARδ WB-Bcl6) was performed with anti- PPARδ followed by western blot using the anti-Bcl6 antibody. The bar graph shows the protein quantification expressed as a percentage. *p<0.05 versus control (ct), §*p*<0.05 versus Dox 0.1, # *p*<0.05 versus L-165141+Dox 0.1. (B) Cytoplasmic extracts were evaluated by western blot with PPARδ and Bcl6 antibodies. (C) The effects of SB203580 (SB), inhibitor of p38, SP600125 (SP), inhibitor of JNK and Akt1/2 kinase inhibitor (Akti), inhibitor of Akt, on the L-165041-induced increases of PPARδ (first line) and Bcl6 (second line). Whole cell extracts were assayed by western blot analysis. All inhibitors reverse the effects of L-165041 on Bcl6 levels, while only the Akt inhibitor reverses the up-regulation of PPARδ. The bar graph shows the protein quantification expressed as a percentage. *p<0.05 versus ct, §*p*<0.05 versus Dox 0.1, # *p*<0.05 versus L-165141.

PPARδ mRNA (data not shown) and protein expression were significantly up-regulated in H9c2 cells treated with L-165041, doxorubicin 0.1 µM or exposed to sequential treatment with L-165041 and doxorubicin 0.1 µM (Fig. 6). Since previous studies indicated that in the absence of a specific ligand, PPARδ may bind Bcl6 [15], [16], we thus examined the effects of doxorubicin and L-165041 on Bcl6 and on PPARδ:Bcl6 interactions by co-immunoprecipitation (Fig. 6A). Whole cell and nuclear extracts revealed that 0.1 µM doxorubicin did not change the expression levels of Bcl6 (Figs. 6A and 6C), but that it did cause a 2-fold increase in the amount of Bcl6 sequestered by PPARδ (Fig. 6A). In contrast, while L-165041, either alone or followed by doxorubicin, increased total Bcl6 (Figs. 6A and 6C) it also decreased the amount of Bcl6 associated with PPARδ (Fig. 6A) thus enhancing the amount of free Bcl6. By using specific inhibitors, we documented that p38, JNK and Akt play a key role in L-165041-induced Bcl6 up-regulation, and that Akt also regulates the L-165041-induced PPARδ up-regulation. In fact, pre-treatment with the Akt inhibitor prevents the increase of PPARδ protein levels in response to L-165041 (Fig. 6C).

These results suggest that L-165041 may counteract the action of doxorubicin through the increased expression and release of Bcl6.

### Bcl6 Plays a Key Role in the Regulation of Senescence in H9c2

**Figure 7:**
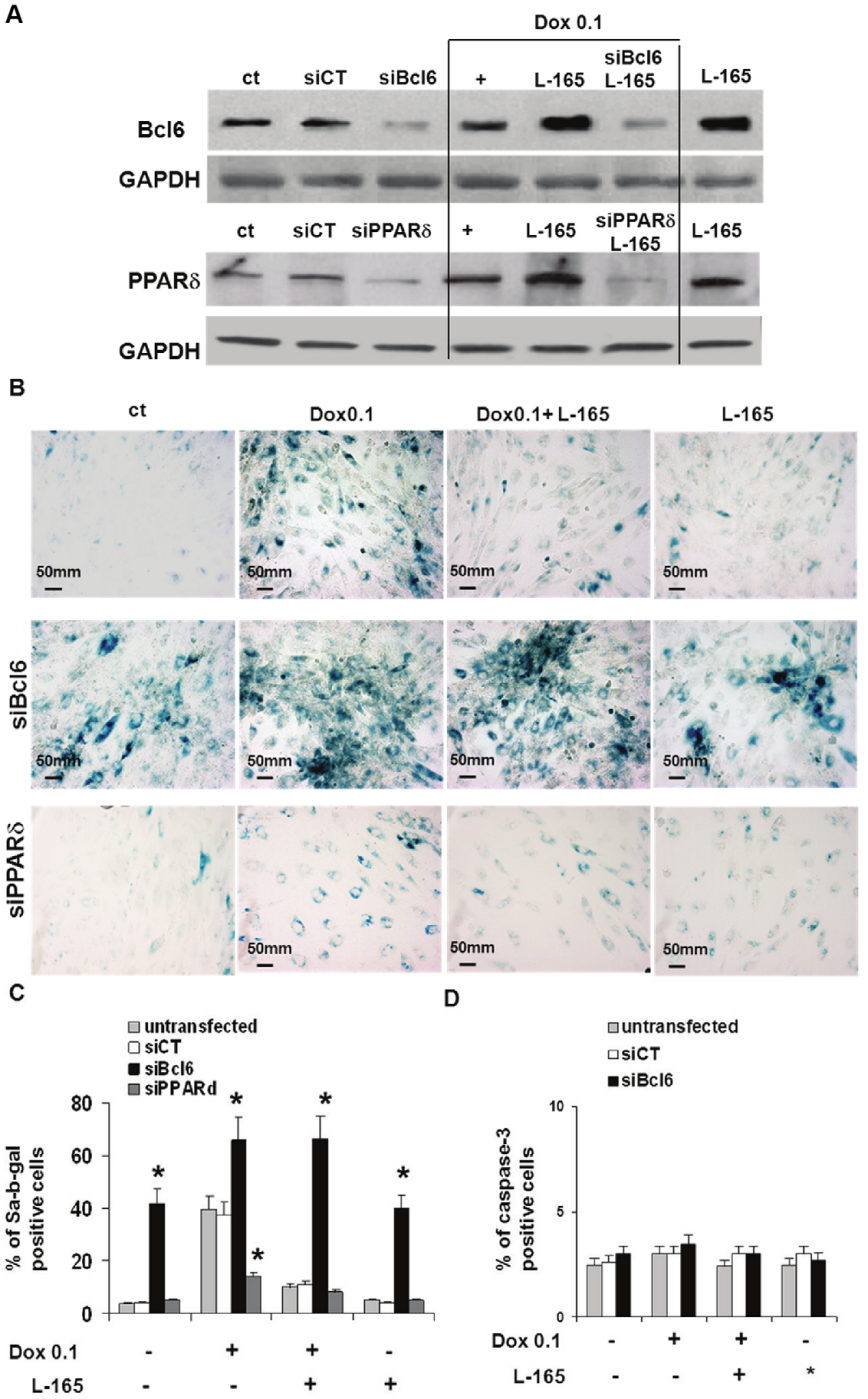
Bcl6 plays a key role in cellular senescence. Bcl6 siRNA(siBcl6) significantly increases the number of SA-b-gal positive cells in both unstressed and in 0.1 µM doxorubicin-treated cells, and it completely abolishes the anti-senescent effect of pre-treatment with L-165041 (L-165). siPPARδ completely abolishes the pro-senescent effect of 0.1 µM doxorubicin. H9c2 cells were transfected with Bcl6-, PPARδ specific or control (siCT) siRNA for 48 h before treatment with or without L-165041 for 2 h followed by treatment with or without 0.1 µM doxorubicin (Dox 0.1) for 3 h. Cells were assayed for protein amount (after 24 h), premature senescence (after 3 days) and apoptosis (after 24 h). (A) Western blot analysis with Bcl6 and PPARδ antibodies. (B) SA-b-gal activity (magnification ×200). (C) Bar graph illustrating the percentage of SA-b-gal positive cells. *p<0.05 versus corresponding siCT of the same treatment condition, (D) Bar graph illustrating the percentage of caspase-3 positive cells.

The results we obtained prompted us to hypothesize that L-165041 might counteract the action of doxorubicin through the increased expression and release of Bcl6. To better understand the influence of Bcl6 and Bcl6:PPARδ interference on doxorubicin-induced senescence, we selectively silenced either Bcl6 or PPARδ using the siRNA transfection technique. We observed that the Bcl6 protein knock-down was associated with a significant increase in the number of SA-b-gal positive cells in both unstressed and 0.1 µM doxorubicin-treated cells, and that it completely abolished the anti-senescent effect of pre-treatment with the PPARδ ligand L-165041(Figs. 7A, 7B and 7C). In contrast, silencing PPARδ remarkably attenuated the pro-senescent effects of doxorubicin (Figs. 7A, 7B and 7C). Control siRNA, consisting of a pool of non-specific sequences, had no effect on SA-b-gal levels (Figs. 7A and 7C). We then became interested in assessing whether silencing Bcl6 could either cause apoptosis in untreated cells or produce a shift in the stress-response program from senescence to apoptosis in cells treated with doxorubicin 0.1 µM. Therefore, we examined the number of cleaved caspase-3-positive cells and we observed that the Bcl6 knock-down did not produce any effects in either untreated or in 0.1 µM doxorubicin-treated cells, with or without pre-treatment with the PPARδ ligand L-165041 (Fig. 7D).

### Activated PPARδ Inhibits Doxorubicin-induced Apoptosis

**Figure 8:**
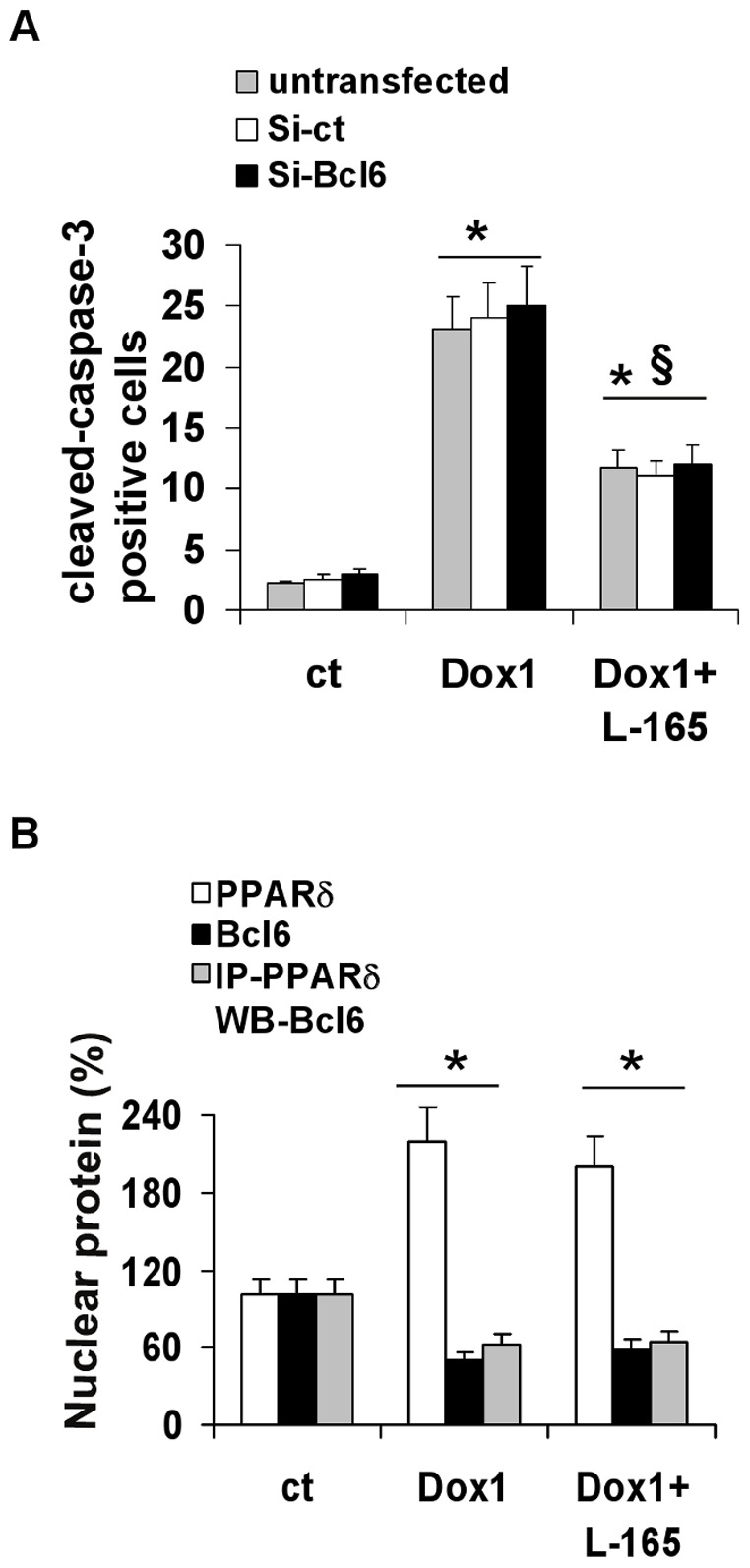
L-165041 protects H9c2 from doxorubicin-induced apoptosis through a Bcl6-independent mechanism. (A) Pre-treatment with L-165141decreases the number of doxorubicin-induced apoptotic cells. Bcl6 siRNA (siBcl6) does not modify the number of activated caspase-3 positive cells in any of the treatment groups i.e., untreated cells, 1 µM doxorubicin-treated cells, and in cells pretreated with L-165041 and then incubated with doxorubicin. 24 h after treatment, cells were assayed by immunocytochemistry for cleaved caspase-3. Bar graph illustrates the percentage of cleaved-caspase-3 positive cells. *P<0.05 versus ct, §*P*<0.05 versus doxorubicin. (B) Effects of L-165041(L-165) and 1 µM doxorubicin (Dox1) on PPARδ and Bcl6 protein expression and on PPARδ and Bcl6 protein interaction. Doxorubicin increases PPARδ levels and down-regulates the amount of total and PPARδ bound Bcl6 (IP-PPARδ WB-Bcl6). Pre-treatment with L-165041 does not modify the effects induced by doxorubicin. H9c2 cells were pre-treated with or without L-165041 for 2 h, then treated with or without 1 µM doxorubicin for 3 h and analyzed after 24 h by western blot. The bar graph shows the protein quantification expressed as a percentage. *p<0.05 versus control (ct).

In the previous paragraphs we reported data demonstrating that pre-treatment with the PPARδ ligand L-165041 prevents senescence induced by doxorubicin 0.1 µM and that this effect mainly occurs through a Bcl6 related mechanism. We further examined the effects of pre-treatment with the PPARδ ligand on cells exposed to pro-apoptotic doses of doxorubicin, and results show that pre-treatment with L-165041 prevents apoptosis induced by doxorubicin 1 µM, as assessed by both A/PI double staining (Fig. 2E) and cleaved caspase 3 (Fig. 8A).

We found that doxorubicin 1 µM produces a two-fold increase in PPARδ expression levels. This increase is not influenced by pre-treatment with the PPARδ ligand L-165041.We also found that doxorubicin 1 µM causes a 50% reduction in both total and PPARδ-co-immunoprecipitated Bcl6. Of note, these changes were not influenced by pre-treatment with the PPARδ agonist (Fig. 8B).

We then examined the effects of transfection with siRNA targeting Bcl6 on apoptosis. We observed that silencing Bcl6 did not increase the apoptosis rate in untreated cells and did not enhance apoptosis in cells treated with doxorubicin 1 µM. We also found that pre-treatment with the PPARδ ligand L-165041 in cells exposed to doxorubicin 1 µM significantly decreased the number of apoptotic cells, and what is noteworthy is that this protective effect was not affected by Bcl6 knock-down (Fig. 8A).

## Discussion

The present study is a step forward towards understanding the cellular mechanisms of doxorubicin-induced senescence and highlights the cardioprotective actions of PPARδ activation.

We showed, for the first time, that pre-treatment with the PPARδ agonist L-165041 is highly effective in preventing doxorubicin-induced senescence in neonatal cardiomyocytes and H9c2 cells. Pre-treatment inhibited TRF2 downregulation and prevented cell cycle changes. It partially rescued cell proliferation blockage, significantly attenuated cytoskeletal remodeling and the early loss of plasma membrane integrity, and significantly reduced the number of cells that were positive for SA-b-gal activity.

We found that both doxorubicin-triggered senescence and the anti-senescent effects of pre-treatment with the PPARδ agonist L-165041 involve the interferences with the Bcl6 repressor. In fact, while doxorubicin 0.1 µM increases the PPARδ protein expression that sequesters the transcriptional repressor Bcl6 in unliganded PPARδ, L-1650141 increases the expression of Bcl6, which upon ligand binding, is released from the PPARδ and is then able to bind to its target genes.

Experiments performed with siRNA analysis techniques very clearly show the key role of Bcl6 in the cellular senescence program. Silencing Bcl6 led to senescence in unstressed cells, potentiated the pro-senescent effects of 0.1 µM doxorubicin, and abolished the anti-senescent effects of pre-treatment with the PPARδ ligand L-165041. By increasing the amount of free Bcl6, PPARδ protein knock-down prevented the prosenescent effects of 0.1 µM doxorubicin.

To the best of our knowledge, this is the first study demonstrating that the transrepressive mode of action of PPARδ plays a key role in the control of cellular senescence. To date, there are very few data on PPARδ, Bcl6 and senescence. By genetic screening, Shvarts et al [23] identified Bcl6 as a potent inhibitor of senescence since it rendered cells unresponsive to anti-proliferative signals from the p19ARF–p53 pathway. Kim et al [24] demonstrated that GW501516, a specific agonist of PPARδ, up-regulates the transcription of antioxidant genes and significantly inhibits Ang II-induced premature senescence of vascular smooth muscle cells. They also found that siRNA-mediated down-regulation of PPARδ markedly suppresses the anti-senescent effect of GW501516, thus suggesting that in their experimental model the agonist-induced PPARδ effects occur without relocation of a repressor.

Unlike the scarcity of data on senescence, there is a large body of evidence showing the role that PPARδ and Bcl6 play in inflammation. PPARδ has been shown to control an inflammatory switch through its ligand-dependent association with, and dissociation from, Bcl6 [15], [17], [27]–[30]. In fact, unliganded PPARδ is pro-inflammatory, while activated PPARδ exerts anti-inflammatory effects [17], [18]. It is not surprising that PPARδ and Bcl6 are involved in both senescence and inflammation since important relationships do exist between inflammation and senescence. It has been shown that Angiotensin II induces vascular inflammation and senescence both *in vitro* and *in vivo* [31]. Senescent cells show a pro-inflammatory phenotype called senescent-associated secretory phenotype because this phenotype is characterized by the secretion of a great deal of inflammatory cytokines which have a profound impact on tissue homeostasis [32]. A tight link between the process of cellular senescence and the IL-dependent inflammatory network has been proven. Using microarray analysis, Shelton [33] et al. demonstrated that senescent fibroblasts present a strong inflammatory type response. Kuilman et al. [34] found that IL-6 is up-regulated in cell lines programmed to prematurely enter oncogene-induced senescence and demonstrated that when IL-6 or its receptor is suppressed, cells re-enter the cell cycle and proliferate. Moreover, clinical studies have documented that some biomarkers of cellular senescence in circulating leukocyte DNA, especially telomere attrition, correlate with incident or prevalent atherosclerotic cardiovascular diseases [35].

We found that p38, JNK and Akt are activated by both the cardioprotective agent, L-165041, and by the cardiotoxic agent, doxorubicin. While Akt activation is generally associated with a protective role [36], p38 and JNK have been identified as stress kinases because they are activated by stimuli that cause some kind of stress to cells which eventually lead to cell death [37]. However, while this assumption is correct in most cases, several studies suggest that activation of p38 [38] and JNK [39] by stress stimuli does not necessarily promote damage, but rather, it enhances cell survival.

Whether MAPK activation executes stress induced damage or survival pathway activation depends on the cell type or type of stress or stimulus. Previous studies on the signal transduction pathway in doxorubicin cardiotoxicity demonstrated that p38 activation is crucial for the execution of doxorubicin-induced damage, while the concomitant JNK and Akt activation has to be viewed as part of a cardiomyocyte survival pathway which attempts to limit the damage caused by doxorubicin [4], [8], [40].

In the present study, we evaluated the mechanism through which agonist-induced PPARδ activation may exert protective effects against doxorubicin-induced senescence. We found that pre-treatment with specific inhibitors of p38, JNK, and Akt prevents the effect of L-165041 on Bcl6 levels and on doxorubicin-induced SA-b-gal, and that pre-treatment with the Akt inhibitor also prevents the effect of L-165041 on the up-regulation of PPARδ. We demonstrated that not only Akt, but also p38 and JNK activation are essential in order for PPARδ activation to exert a protective effect. This is in agreement with both the study by Liang et al. who demonstrated that L-165041 inhibits C-reactive protein induced inflammation in cardiomyocytes and in H9c2 through p38 and JNK [41] and with the study by Yue et al [42] who found that PPARδ activation enhances Akt signaling and protects the heart from ischemia/reperfusion injury in Zucker fatty rats.

We also found that pre-treatment with L-165041 prevents the doxorubicin-induced increase in pJNK and pAkt but not the doxorubicin-induced increase in pp38. It is possible that the protection provided by L-165041 through Akt and JNK signaling is able to prevent doxorubicin-induced stress so that doxorubicin does not cause any further activation of these survival pathways. Protection through the activation of p38 occurs with an initial increase in phosphorylation due to pre-treatment with L-165041, followed by a further increase in phosphorylation due to treatment with doxorubicin.

Collectively, our data show that Bcl6 plays a main role in the protective effect exerted by L-165041 against doxorubicin-induced senescence: L-165041 increases Bc16 expression levels through p38, JNK and Akt mediated pathways and induces its release from PPARδ thus allowing Bcl6 binding to its target genes to exert its anti-senescent actions.

Although apoptosis was not the main issue of our study we repeated several experiments using doxorubicin 1 µM, i.e., a pro-apoptotic dose, to compare the role played by the PPARδ agonist in senescence and apoptosis. We found that pre-treatment with the PPARδ agonist L165041 is effective in preventing apoptosis induced by doxorubicin 1 µM. Even though Bcl6 was down-regulated by doxorubicin, RNA interference experiments documented that it is neither implicated in the execution of doxorubicin-induced apoptosis nor in the anti-apoptotic effects exerted by pre-incubation with the PPARδ agonist. Studies investigating the role of Bcl6 in apoptosis produced inconsistent results [43]–[48]. Since doxorubicin-induced apoptosis is largely reactive oxygen species mediated [49], we speculate that upon ligand binding, PPARδ is enabled to induce transcription of genes encoding the antioxidant enzymes. This hypothesis is in agreement with previous studies by Pesant et al, who found that the PPARδ agonist GW501516 protects H9c2 from H2O2-induced cell apoptosis. They also found that this protection is totally dependent on PPARδ and is carried out through catalase up-regulation [26]. In addition, since it has been shown that PPARδ agonists also enhance the physical interaction between PPARδ and the p65 subunit of NF-kB, thus preventing its ability to induce gene transcription [29], [50], it can be hypothesized that even this mechanism might contribute to protect cardiomyocytes from the pro-apoptotic effects of doxorubicin.

It is also worthy of note that silencing Bcl6 in cells treated with doxorubicin 0.1 µM potentiated the cardiotoxic effects of doxorubicin by increasing its pro-senescent effects without inducing a switch to apoptosis. The fact that Bcl6 is crucial for senescence induced by doxorubicin 0.1 µM, but not for apoptosis induced by doxorubicin 1 µM confirms that senescence and apoptosis are two very distinct stress response cellular programs.

Since the most functionally significant cell type in the heart is represented by post mitotic, terminally differentiated cardiomyocytes, the idea of investigating both anthracycline cardiotoxicity and PPARδ activation cardioprotection by studying mechanisms of cellular senescence in dividing neonatal rat cardiomyocytes and H9c2 might seem, at first glance, odd. It must be said however that this model has been extensively used in the past and it has been considered a convenient approach for preliminary investigations [3], [4], [51]. In addition, in very recent years, convincing evidence has shown that the normal heart is not a post mitotic organ since it contains a pool of progenitor cells and a population of immature, dividing myocytes that allow for a turnover of cardiomyocytes involving the generation of new cardiomyocytes in substitution of the damaged ones [52]. A new view on anthracycline cardiotoxicity was recently introduced with the demonstration that in comparison to differentiated cardiomyocytes, dividing cardiomyocytes are more sensitive to anthracyclines and that low doses of doxorubicin causes senescence-like changes in these cells [3], [4], [53], [54].These effects may inhibit the regenerative capacity of the heart and, through this mechanism, impair the self-repairing potential of the heart, ultimately leading to ventricular dysfunction and late clinical events. Neonatal cardiomyocytes share some characteristics with the population of replicating cells that are present in the adult heart, and that are reminiscent of a fetal/neonatal phenotype [52]. Thus, this experimental model may be considered a convenient indicator of what might happen to these cardioregenerative cells when the heart is exposed to anthracyclines with or without.

pre-treatment with a cardioprotective agent. The work presented here demonstrates for the first time that the PPARδ activator L-165041 is a highly effective cardioprotective agent, and suggests that further testing including animal models of anthracycline cardiac injury should be carried out to assess the potential therapeutic role of this compound.

## Materials and Methods

All materials, unless otherwise indicated, were supplied by Sigma-Aldrich (Poole,UK).

### Cell Culture and Treatment

Neonatal rat ventricular myocytes (Clonetics® Rat Cardiac Myocytes, Lonza, Switzerland) were purchased and cultured with rCMC Medium Bulletkit (Clonetics, Lonza, Switzerland) as described [55]. H9c2 rat heart-derived embryonic myocytes (American Type Culture Collection) were cultured as previously described [9]. Cells were always used at less than 70% of confluence.

*Experimental design:* Cells were pre-incubated for 1 hour with or without the ERK1/2 pathway inhibitor PD98059 (50 µmol/L) (Calbiochem, Merk, Germany), the JNK inhibitor SP600125 (20 µmol/L), the p38 MAPK inhibitor SB203580 (3 µmol/L), with the Akt pathway inhibitor Akt1/2 kinase inhibitor (30 µmol/L), and then were incubated for 2 hours with or without L-165041 (10 µmol/L) (Calbiochem, Merk, Germany). They were then treated with or without various doses of doxorubicin for 3 hours [4] and analyzed at the time indicated for each experiment. In order to evaluate MAPK and Akt phosphorylation, cells were pre-treated for 20, 40, or120 minutes with or without L-165041, then they were treated for 20, 40, or 120 minutes with or without doxorubicin. Since both L-165041 and the MAPK inhibitors were dissolved in 0.1% dimethyl sulphoxide (DMSO), an equivalent amount of vehicle was added to both the control and to the drug-treated samples when the experiments were performed with these inhibitors.

### Semi-quantitative Reverse Transcription PCR

RNA isolation and RT-PCR were performed using the previously described procedure [56]. The primers used for PCR were TRF2 forward (GGACAGCGAGACACTAAAGGC), reverse (CTGGATGACAATGTCTG CTTC) GenBankTM accession number NM031055. PPARδ forward (AGGCCTCA TGAACGTGCCACAG), reverse (AGCGGACTAATGGGCTCACTGG) GenBankTM accession number NM013141. GAPDH forward (GAGGGGCCATCCACAGTCTT), reverse (TTCATTGA CCTCAACTACAT), GenBankTM accession number XM221254.

The quantity of mRNA was normalized for Glyceraldehyde-3-Phosphate Dehydrogenase (GAPDH). The PCR products were quantified by a gel documentation system with analysis software (Syngene, Cambridge, UK).

### Quantitative Real Time PCR Analysis

Total RNA was extracted from 6-wells of cultured cells from neonatal rat ventricular myocytes using RNeasy mini Kit according to the manufacturer’s protocol (Qiagen, Germany). Twenty µl of total RNA were reverse transcribed using the High-Capacity cDNA Archive Kit (Applied Biosystems by Life Technologies Foster City, CA, U.S.A.) and pre-amplified using the PreAmp Master Mix KitPrimers, and probes for PPARα, PPARγ, PPARδ and GAPDH were obtained from a pre-developed assay-on-demand (Applied Biosystems by Life Technologies Foster City, CA, U.S.A.).

Five µl of the resulting pre-amplified, cDNA dilution (1∶2) were used for quantitative PCR amplification which was performed in quadruple on the Prism 7900HT Instrument (Applied Biosystems, Foster City, CA, U.S.A) with the fluorescent TaqMan method. The PPAR mRNA quantities were normalized to the control gene GAPDH and were expressed in relation to a calibrator sample. The levels of transcripts PPAR and GAPDH were determined using the standard curve. The standard curve was obtained with serial dilutions (106-10 molecules) of a cDNA calibrator.

### Total Cell Lysates

Cells were lysed in lysis buffer [20 mM Tris HCl (pH 7.5), 150 mM NaCl, 1 mM Na2EDTA, 1 mM EGTA, 1% NP40, 2.5 mM Na2P2O7, and 1 mM β-glycerophosphate]. The following inhibitors were added immediately before adding the buffer to the cells: 1 mM phenylmethylsulfonyl fluoride, 1 mM Na3VO4, 1 mM NaF, and protease inhibitor mixture (Roche, Switzerland).

### Subcellular Fractionation

Cytoplasmic and nuclear fractions were obtained as previously described [57]. The nuclear fractions were supplemented with 50U endonuclease benzonase to obtain both the soluble and the chromatin-bound insoluble pool of nuclear proteins.

### Co-immunoprecipitation

To examine the interaction between PPARδ and Bcl6, the nuclear protein fraction was co-immunoprecipitated with the anti-PPARδ antibody as previously described [17] and then analyzed by western blot using the anti-Bcl6 antibody.

### Western Blot

Immunoblotting was performed using the previously described procedure [9]. After various treatments, cells were processed to determine the levels of TRF2 (clone 4S794.15, Imgenex, USA), PPARδ (ab23673, Abcam USA**)** phosphorylated p38 (T180/Y182 site, R&D Systems) Bcl6 (D-8) GAPDH (0411), p38 (H-147), phosphorylated JNK (G-7) (pJNK) and JNK (FL), phosphorylated Akt (pAkt) (ser473)-R, Akt (H-136), phosphorylated ERK1/2 (pERK1/2) (E-4), ERK1/2 (K-23) (Santa Cruz Biotechnology, Santa Cruz, USA). After incubation in horseradish peroxidase secondary antibody, blots were visualized with ECL substrate (Millipore, Merk, Germany) and films were quantified by densitometry using an image analyzer system (Syngene, UK). Filters were probed with GAPDH in order to normalize the amounts of TRF2, PPARδ and Bclδand with p38, JNK, Akt, ERK1/2 antibodies to normalize the amounts of pp38 and pJNK, pAkt, pERK1/2, respectively. The immunoblots of nuclear extracts were hybridized with anti-TATA binding protein antibody (Abcam) to monitor equivalent loading in different lanes.

### SA-b-gal Activity (Senescence-associated -β-Galactosidase Staining)

Cells were stained for β-gal activity as described by Dimri [58].

The ability to induce SA-b-gal activity is a manifestation of residual lysosomal activity at suboptimal pH (pH 6). It becomes detectable in the course of senescence because of the increased lysosomal content on senescent cells [59]. The number of SA-b-gal positive cells was determined in 100 randomly chosen, low-power fields (x100) and expressed as a percentage of all counted cells.

We chose to analyze the level of senescence three days after the end of the treatments because, had it been analyzed earlier, the percentage of senescent cells would have been too low to allow statistical analysis.

### MTT Assay

The assay, which is based on the reduction of the tetrazolium salt MTT by active mitochondria to produce insoluble formazan salt, measures mitochondrial metabolic activity and is often used as an indicator of cell viability. Cells were treated in 96-well plates, MTT was added to each well under sterile conditions (final concentration of 5 mg/ml), and the plates were incubated for 3 h at 37°C. Formazan crystals were dissolved in dimethyl sulfoxide (100 ml/well). The purple formazan crystals were formed from yellow MTT by succinate dehydrogenase in viable cells. Absorbance of the formazan product was measured at 570 nm with a background correction at 690 nm using a microplate reader [60].

### Annexin V–fluorescein Isothiocyanate (FITC)/Propidium Iodide Staining

Cells were labeled with AV/PI, and 100 randomly selected fields were counted using a fluorescence microscope. The number of stained cells was normalized to the total number of cells as counted by phase contrast microscopy of the same field.

### Immunocytochemistry

Expression of cleaved caspase3 protein was documented by immunostaining using the Cleaved Caspase-3 (Asp175, Cell Signaling) antibody and was performed with the procedure described elsewhere [61]. This antibody detects endogenous levels of the large fragment (17/19 kDa) of activated caspase-3 resulting from cleavage adjacent to Asp175. Caspase-3 is a critical executioner of apoptosis. Activation of caspase-3 requires proteolytic processing of its inactive zymogen into activated p17 and p12 fragments. Cells were examined by light microscopy for image analysis.

### Flow Cytometric Analysis

Trypsinized and floating cells were pooled, washed twice with PBS and resuspended in 400 µl of hypotonic labeled solution, 5 µg/ml PI, 0.1% w/v Na citrate, 0.1%Triton X-100 in sterile water. Cells were incubated on ice for 30 min. until DNA content analysis.

Both nuclear DNA content and cell cycle analysis were monitored by Fluorescence Activated Cell Sorting (Becton Dickinson, San Jose, CA) and data were analyzed using CellQuest software (Becton Dickinson San Jose, CA).

### F-actin Detection

Cardiomyocytes growing on slides were fixed, permeabilized and labeled simultaneously in PBS containing 50 µg/ml lysopalmitoyphospatidylecholine, 3.7% formaldehyde and 5 units/ml of fluorescent phallotoxin (A-12379alexaTM488 phalloidin, Molecular probes, Inc). Cells were rapidly washed three times with PBS and were viewed by fluorescent microscopy [62] in order to carry out image analysis.

### Bcl6 and PPARδ siRNA Transfection

ON-TARGET plus SMARTpool short interfering RNAs (siRNA) for silencing the expression of target genes Bcl6, PPARδ and ON-TARGET*plus* Non-Targeting Pool as a control were purchased from Dharmacon (Thermo Ficher Scientific, USA). All transfections were carried out according to the manufacturer's instructions with DharmaFECT1 transfection reagent (Thermo Fisher Scientific, USA). Briefly, cardiomyocytes were trypsinized, counted, and plated at a density of 104 cells/cm2. After 24 hours, cells were transfected with 100 nmol/l of SMARTpool siRNA or control siRNA using DharmaFECT1 reagent and analyzed after 24, 48 or 72 hours by immunocytochemistry for caspase 3, SA-b-gal activity and Western blot analysis.

### Image Analysis

Image analysis was performed by the Leica Q500 MC Image Analysis System (Leica, Cambridge, UK). Three hundred cells were randomly analyzed for each sample, and the optical density of the signals was quantitated by a computer. The video image was generated by a CCD Camera connected through a frame grabber to a computer. Single images were digitized for image analysis at 256 grey levels. Imported data were quantitatively analyzed by Q500MC Software-Qwin (Leica, Cambridge, UK). The single cells were randomly selected by the operator by using the cursor and then positive areas were automatically estimated.

### Statistical Analysis

Data are reported as mean±standard error of four independent experiments. Statistical analysis was performed by one-way ANOVA followed by the Bonferroni post-hoc test and by the Wilcoxon signed rank test when appropriate.
